# Part III. Clinical Review and Hospital Reports

**Published:** 1834-04-01

**Authors:** 


### Clinical Review.

### I. Remarks on some frequent forms of Venereal Ulcer. By Mr. Henry James Johnson, formerly House-Surgeon to the Lock Hospital.

There is a form of venereal ulceration of which I have witnessed numerous examples—which may therefore be considered as not uncommon—which displays decided and peculiar characters—which runs a marked and not indefinite course—which readily yields to one method of treatment—and, so far as I have seen, resists most others.

I am unacquainted with a good description of this venereal ulceration, and the one I am about to offer is taken from the cases I have noticed. Before I enter on it, I shall venture to indulge in a few introductory remarks.

The venereal disease has been frequently studied in an unsatisfactory manner. The majority of writers would seem to have laboured to establish or to overturn a theory. Their principal aim would appear to have been to invent some ideal, or to erect some actual sore into the true type and symbol of syphilis, and to view the various ulcers, the existence of which even they could not deny, as only the legitimate or the illegitimate offspring of that parent stock.

Mr. Hunter fancied or found a sore which he seems to have considered the genuine syphilis. Why he selected that ulcer for that honour it would be idle, perhaps it would be vain, to inquire. The example of Mr. Hunter has not been lost. His successors in this department of science have been blinded by his authority and confounded by his error. Those who have supported and those who have opposed him have been equally affected by his perplexing hypothesis. On the one hand we distinguish a large class of writers who have toiled to prove that mercury is unnecessary, even for the true Hunterian chancre. On the other hand we perceive as numerous a body contending for the utility, indeed for the necessity, of mercury in this case, but yielding the other forms of ulcer, as indeterminate and bastard maladies. It would probably be invidious to particularize authors or their works, but a moderate acquaintance with the latter is sufficient to convince a reflecting and unprejudiced reader that, in one way or other, they are greatly influenced by the doctrines of John Hunter.

It is difficult for any one who enjoys opportunities of observing the venereal disease extensively, to shut his eyes to the obvious fact, that if the Hunterian chancre is to be deemed the only genuine syphilis, that malady has almost ceased to exist. Yet secondary symptoms are sufficiently frequent to shew that if syphilis is gone, specific sores are left in its place, followed by the same consequences and requiring the same treatment.

When we turn from the doubtful field of speculation to contemplate the objects that nature presents, we are struck at first by their apparent intricacy. In a hospital devoted to the reception of patients affected with the venereal disease, the crowd of cases perplex the observation and confound the judgment. The varieties of sores appear interminable, and probably out of a lengthened series none exhibit features of identity. The discordance between ideas derived from reading and the facts displayed before his view, commands the attention and excites the reflection of the observer. That discordance is too striking and too irresistible to permit him to avoid the inevitable conclusion, that a practical, to say nothing of a profound, acquaintance with this singular disease, can only be obtained by personal labour and personal investigation.

The extensive application of the inductive method of philosophy to medicine has been of comparatively recent date. Yet its triumphs have been splendid and must be permanent. The striking advances of the present day in physiology, pathology, and therapeutics, have been solely, or almost solely, due to the cultivation of the inductive method.

That method is remarkably adapted to the investigation of a malady like syphilis. The observer has numerous and complicated phenomena displayed before his view. Patient observation and rigorous induction can alone enable him to unravel their intricate arrangement, and discern their actual relations. Like the clue of Ariadne it leads him through the maze.

There are sores of different descriptions. Some appear as individuals—some are arranged in clusters, or produced in numbers. Some are small—some large. Some are attended or succeeded by an indurated basis—others display but an inconsiderable tumefaction. Some present a healthy aspect—others are yellow on the surface—and others exhibit all the shades of difference that separate a simply sloughy appearance from that of actual sphacelus.

The cicatrices differ like the sores. One resembles that of an ordinary ulcer. Another is the seat of redness and of induration. A third evinces an incessant tendency to excoriation. A fourth re-ulcerates and forms again a sore.

The ulcers of the throat are as complicated and as various as the primeval sore. On the one hand we remark a trifling vascularity of the tonsils, the palate, and the pharynx. On the other, we contemplate a frightful ulcer, destroying, rather than occupying, these important parts. Between the two extremes are a yellow ulceration inclined to be deep, or surrounded with thickening — a superficial white ulceration with enlarged and indurated tonsils—a brown and sloughy ulceration of these bodies—and other, less important, diversities of character, to which it would be useless to allude.

The affections of the skin may occupy almost all the recognized classes of cutaneous alterations. The papula—the efflorescence—the stain—the vesicle—the small and large pustule—the crust—the irregular thickening—the tubercle—and scale, are constantly presented in the wards of the Lock Hospital.

The man who is calculated to throw a brilliant or a steady light on the venereal disease, is one who is patient in observing, cautious in concluding—who is sceptical of even what he sees, and admits nothing which is not established by the strictest rules of evidence.

His field of observation must be ample. He must watch the sore in all its stages—observe its varying features—and mark the cicatrix that it leaves. He must note the secondary symptoms that succeed (*if* secondary symptoms follow)—their character, their combinations. He must study the influence of diet and of medicine, especially of mercury. He must note with accuracy that which he observes with care. A few years consumed in such observation and reflection would enable a man of moderate ability and of good acquirements to confer a great benefit on himself, the profession, and the public, by the general dissemination of sound opinions and judicious practice.

In the mean time, those who are possessed of the means of observation may occupy themselves without discredit, in drawing attention to special facts, and in laying before their professional brethren, in journals and in societies, the conclusions they have formed or the cases they have seen. Actuated by this feeling, which I hope is not improper, I have ventured to request attention to the sores I shall now proceed to describe. Perhaps an apology is due for the length of the preceding observations. I might say with the learned and ingenious Selden, "had my time been longer, my remarks would have been shorter."

*Situations*. The sore to which I have alluded is usually remarked on some portion of the inner prepuce. Perhaps the most frequent situation is the angle, or that part where the skin is reflected from the prepuce to behind the corona of the glans.

Next in order of liability is the orifice of the prepuce, when naturally narrow.

The side of the frænum is often affected; the inferior surface of the penis, and contiguous scrotum, are occasionally implicated.

I shall not describe its history in women.

It is sometimes complicated with gonorrhœa, but commonly it exists independently of this affection.

When seated *at* the angle, or *on* the inner prepuce, it frequently produces inflammatory phymosis. This is commonly developed within the first week.

When situated at the orifice, it causes phymosis also, but not in a similar manner. In this case the phymosis is the result of induration occasioned by the sores, is developed later, and is usually disposed to continue longer.

The sore is seldom single. It may commence in a solitary manner, but others always form, and they often become confluent.

It appears in its commencement as a pustule or a vesicle, beneath the epithelium. In a very short time, perhaps in a few hours, the cuticle at the apex is absorbed, and a small yellow surface, about the dimensions of the head of a pin is presented.

This increases in size till it reaches the diameter of a split pea, which perhaps it may exceed. The surface is then yellow and cupped—the edge is thin and slightly undermined—there is trifling surrounding redness—and some subjacent tumefaction.

Four or five days are commonly consumed in attaining this condition. It may remain stationary for many days more, but usually the following changes supervene.

Granulations, at first minute, but afterwards distinct, appear upon the surface. They are spongy and yellow. They often surmount the level of the skin.

The edge of the ulcer becomes distinct, and a vascular line is observed within it. This is the precursor of cicatrization.

Previous to the completion of the latter, the sore is generally florid, frequently raised, and almost always accompanied with induration.

*The cicatrix* is red and generally hard.

The sore is invariably circular, but many forming near each other, always become confluent, and produce an irregular ulcer.

The duration of the sore appears indeterminate. The ulcer may remain, if medical treatment is withheld, for an almost indefinite period. At all events I am unacquainted with its limits.

A distinguishing feature of these sores is their number. Whilst one is passing through the stage of granulation, another is making its appearance.

This constant succession of fresh sores, or fresh crops, continues till the final cure.

Sometimes the sores, instead of arising as separate vesicles, appear as one continuous, sloughy-looking vesication, occupying the whole circumference of the angle.

The sore is contagious in a high degree. Opposite surfaces infect each other. The lodgment of the discharge is certain to give rise to it. I have recently had under my care a married couple. The husband infected the wife.

*Bubo.* Bubo is frequently noticed. It sometimes proceeds to suppuration, but with care this unpleasant consequence may be commonly prevented.

*Secondary Symptoms.* I have found ulceration of the throat, and eruptions of three characters succeed this sore.

In one patient at the Lock, I remarked a yellowish white ulceration of the tonsils—attended with an eruption of papulæ pustulating at their summits.

In another there was yellowish ulceration of the tonsils, and eruption of small and scaly spots, approaching to the small psoriasis.

In a third the eruption was the syphilitic lepra.

I cannot enter on the reasons which lead me to point these out as peculiarly apposite examples of true venereal eruptions.

*Treatment.* The first stage of this sore is one of inflammation—the second is that of granulation and repair. They display different phenomena—require different treatment.

The remedies at first should be antiphlogistic — purging — rest — starvation. Locally, bread poultices and tepid washes, or cold and saturnine lotions.

I usually prescribe three grains of calomel at night and the senna draught on the succeeding morning, accompanied, if necessary, with salines during the day.

As soon as granulations have appeared, and the red line, denoting a tendency to cicatrize, has become developed at the margin, the treatment must be altered.

Moderately increase the patient's diet. Diminish the purgation—commence a course of mercury—use stimulating applications to the sore — and touch it every second or third day with the lunar caustic.

I commonly order three to five grains of blue pill twice daily, and the infusion of roses and salts every second or third morning.

The best local applications, are, *ablution* of the parts twice daily with the Bates's red wash, and the constant application of the calamine cerate.

This plan should be continued for a fortnight, or thereabouts, when, probably, the purgative may be discontinued, and certainly the sarsaparilla may be usefully prescribed.

The patient *must* go through a course of mercury to ensure his safety. Five grains of blue pill twice daily are probably the utmost quantity, and four or five weeks the time that he requires.

I shall now take the liberty of relating some cases illustrative of the main features of the foregoing remarks. The points of most importance and the most characteristic of this species of sore, are—plurality in number and succession in appearance—disposition to fungous granulations — their contagious properties — their secondary symptoms —and the influence of treatment. The cases which follow are intended to display and to elucidate these characters. The fear of appearing to indulge in repetitions, compels me to curtail their number and extent.

Case 1. *Plurality of Sores—appearing in Succession—Cure.*

A respectable man, aged 25, applied to me on the 14th of January last.

The inner prepuce was generally red and inflamed. On the left side were several sores—three rather larger than a pin's head, clustered—one, about the size of a split-pea, solitary; on the right side was one of about the dimensions of two split-peas.

The surface was yellow, and just attaining the level of the surrounding skin —the edges were thin, sharp, beginning to be raised, and slightly red.

Tongue whitish—bowels rather confined—pulse frequent—skin warm.

He had had connexion with a stranger nineteen days before I saw him. He had noticed the sores for seven days. He had had no medical treatment.

*Hyd. sub.* gr. iij. *hâc et crast. nocte.*

*Haust. sennæ seq. mane.—Lot. nig.*

21*st*. In a day or two after last report the yellow appearance of the sores diminished, and minute red granulations appeared. These have increased, and the sores have become elevated *above* the level of the neighbouring skin; they have also become confluent both on this and on the right side. Their surface is more florid and their edge red. A fresh sore, about the size of two pins' heads, has appeared on the right side; it is cupped and yellow.

*Pil. hyd.* gr. v. *o. n.*

*Infus. ros.* c̄. *Mag. sul. o. m.*

*Lot. rub. vice Lot. nigræ.**

*Argenti nitras ulceribus elevatis.*

24th. Sores healing—duller vascularity around, and appearance of thickening in the surrounding skin and cellular membrane. On the right side the yellowness has not yet been superseded by vascularity, nor the hollowness by elevation.

*Rep. haust. Mag. sul. o. a. m.*

*Argent. nit. ulc. elevatis.*

28th. Bowels rather relaxed.

*Rep. Pil. bis die, addendo Ext. conii,* gr. ij

*Omr. haust. purgans.*

*Arg. nit. o.* 3*tio die ulc. elevatis.*

*P.* c̄ *Lot. et Cerat. calaminæ.*

30th. Sores on left side healed—each with a white thickened edge immediately surrounding a minute central cicatrix —some subjacent and surrounding tumefaction and induration. On the right side, the sore is almost healed, with the same thickened edge, and immediately within that, a vascular healing line. Gums scarcely affected, health good. *P.*

*To take some meat for dinner daily.*

Feb. 3. Rather a disposition to fresh superficial ulceration of sores—looks pale and thin—pulse frequent—bowels a good deal purged.

*Adde Opii,* gr. ½ *pilulis.*

*Dec. Sars. c.* ℥ij. *bis die.*

10th. Sores healed, with the exception of one no larger than a pin's head, in the angle on the left side. In site of all some thickening. Health much improved. He requires some salts occasionally to retain his bowels in good order.

*To take half a pint of porter daily.*

13th. Sore last noticed healed—fresh superficial ulceration on both sides. Gums not sore. I may state, once for all, that these fresh ulcerations always occurred in the following manner. The inner prepuce presented some redness. In the centre of this, a minute elevation of the cuticle was noticed, which, under the lens, appeared to be a vesication. If this was observed on one day, a small yellow sore had occupied its situation on the next. In the early period of the case, this yellow sore increased in size, grew cupped, and was attended with a thin, sharp edge. Then minute red granulations appeared, giving the yellow sore the appearance of having been sprinkled with a few minute grains of Cayenne pepper. The next stage was universal granulation, of a yellowish-red tint, elevation, and, under the influence of treatment, cicatrization, accompanied with surrounding and subjacent induration.

But the fresh ulcerations that arose in the advanced condition of the case differed, in these respects, from the foregoing type:—all the stages were accelerated; the little yellow ulcer appeared sooner—was less cupped—at-

* The Lotio Rubra is the Camphorated Wash of Bates.

tained less size—presented no fungus—healed with rapidity—and left trifling induration. The application of the nitrate of silver on the second or third day seemed to stop the progress of the sore, and induce its immediate cicatrization.*

I need not pursue the details of the case, as it passed progressively and steadily to a cure. The local treatment was pursued with regularity, until no fresh ulcerations were presented, a period of about a week from the last report. The mild mercurial ointment was then introduced, and retained between the prepuce and the glans. The blue-pill was persevered in till the last day of February when it appeared no longer requisite, and was finally discontinued. The sarsaparilla was taken for ten days after the completion of the mercurial course.

Before the mercury was given up, the induration of the sores had disappeared. The cicatrices were neither tumefied nor red, but some general redness of the inner prepuce remained. The health was good—as good as it had been before the contraction of the malady; the patient, indeed, affirmed that it was better. The gums were gently, and but gently, affected.

CASE 2.—*Plurality of Sores—Bubo—Cure.*

W. Morris, æt. 19, a groom, was admitted an out-patient on the 17th Aug. 1833.

There were numerous sores in the angle, and on the opposed surfaces of the prepuce and corona—they were chiefly disposed near the frænum on each side. The sores were generally yellow, irregularly circular, extending through the cutis, rather flat upon their surface; the edges were abrupt and undermined. The discharge was profuse and purulent. There was much swelling and some inflammation of the prepuce.

A bubo of some size, and rather tender, in the right groin.

Looks pale—tongue moist—bowels rather confined.

He has had the sores for six weeks. The complaint began as a simple sore on the right side of the frænum; the others have appeared in succession. He seems to have taken little or no mercury.

*Hyd. submur.* gr. iij. *hâc nocte.*

*Inf. ros.* c̄ *Mag. sulph.* ℥j. *bis die.*

*Lot. nigra ulceribus.*

20th. Sores cleaner.

*Ung. hyd.* ʒj. *o. n.—Mag. sul. p. r. n.*

*P.* c̄ *Lot.—Omr. alia.*

*To eat some meat daily.*

24th. Frænum ulcerated on its surface—sores still yellow, not granulating, discharging copiously. Looks pale—pulse weak.

*P. To take half a pint of porter daily.*

26th. Looks pale, and rather anxious—skin warm, pulse very frequent. Præputial sores granulating—those on the opposed glans still yellow, irregular, excavated.

I felt convinced that I had committed an error in putting the patient on mercury and on tonics so soon. From his aspect, and the pyrexia he displayed, I apprehended that some unpleasant consequence would supervene. I ordered him to discontinue the mercury, the meat, the porter, and place himself on the following plan.

*Hyd. sub.* gr. iij. *Opii,* gr. ¼. *hâc nocte.*

*Mist. Camph.* ℥j. *Tinct. hyos.* ʒss. *Vin. Ipec.* m. vj. *M. ter die sum.*

28th. Better in all respects—pulse quiet—skin moist and cool—anxious aspect gone. Vascular healing edge to *all* the sores. *P.*

30th. Sores improving, two of the smaller being healed. No pyrexia.

*Pil. hyd.* gr. v. *o. n.*

*Infus. ros.* c̄ *mag. sul. o. a. n.*

*Lot. rubra et cer. calamin.*

*Omr. alia.*

Sept. 3d. Old sores healing with rapidity. Two or three fresh ones, of the characters now so frequently noticed,

---

* This description may be considered unnecessarily prolix. Observation, to be accurate, must be minute; and it is not possible to convey in a few words the distinctions of small particulars.

have appeared upon the inner prepuce. *P.*

7th. Almost all the old sores are healed. Those which arose last are in the state of granulation.

9th. One fresh sore to-day—others all but healed.

14th. Gums scarcely affected.

*Rep. pil. bis die et haust. bis in hebdomadâ tantummodò.*

*To take some meat daily.*

Oct. 5th. Sores quite well for some days. There is still slight induration and redness of the inner prepuce.

19th. The appearance of the prepuce is perfectly natural. His health is good, and he has gained flesh.

*Dec. sars. c.* Oss. *quotid. in hebdomad. Omr. alia.*

This patient has remained free from syphilitic symptoms. He has lately been under my care for gonorrhœa.

The preceding case displays a difficulty of no mean character in the treatment of venereal sores. I allude to the determination of the time when mercury or tonics can be usefully commenced. As a general rule, it is well that the stage of granulation shall begin, and that all inflammatory symptoms have subsided, before the former is exhibited. The general condition of the patient is the guide for the selection of the latter. Perhaps there is no part of the management of syphilis, in which the incautious or the injudicious surgeon breaks down so often and so fatally as in this.

It will be noticed that caustic was not used in this instance. It is of consequence to recollect this, because it has been said that caustic or excision are indispensably required to remove these sores. The former is always beneficial, sometimes absolutely necessary. The latter I would neither submit to, nor inflict.

Case 3.—*Plurality of Sores of the Inner Prepuce and Glans—Mercury insufficient, unaided by Caustic, &c.*

I was requested by my friend, Mr. Roney of Suffolk Place, to meet him in consultation on the following case. I will state the condition of the patient when we saw him, relating the previous and the subsequent particulars in the words of Mr. Roney, who has kindly sent them for that purpose.

The patient, Mr. W. G., was twenty years of age, and of a delicate constitution, having suffered much from eruptive complaints. I had the pleasure of seeing him with Mr. Roney on the 20th of February last.

The prepuce naturally covered the glans. On retracting it several sores were disclosed on the inner prepuce *near* and *at* the angle—there were also some on the opposed glans.* Those on the inner prepuce were circular or oblong, yellowish, elevated, with spongy granulations. Those on the corona and the glans were yellow, scarcely raised, and with a thin sharp edge. On the left side of the glans, near the orifice of the urethra, was one circular solitary ulcer, of the size of a small split-pea, cupped, with a thin undermined edge, and totally void of granulation.

The glans and the inner prepuce were generally red and slightly inflamed. The discharge from the sores was purulent and copious.

No bubo.

The mouth was under the influence of mercury; the bowels were freely open.

He was first seen by Mr. Roney on the 27th of December. The following is that gentleman's account of the case.

" Eight days previously to my seeing him he began to feel a slight itching about the glans on the fourth day after connexion. He was not uneasy, and did not examine it until the succeeding day, when he observed a pimple deeply coloured, with a head which had the appearance of being about to burst. When I saw him there was a small sore not quite the size of a split-pea, situ-

* When one of these sores is found on the inner prepuce, another is tolerably sure to appear on the part of the corona or the glans exactly opposite. The one surface infects the other. The whole progress of these sores shews the highly contagious or infectious properties of their surface and their discharge.

ated in the fold between the glans and prepuce, rather inclining more towards the former; its edges were elevated, and there was considerable inflammation around. He complained a good deal of pain, at times much more severe than others, as if he frequently received a sting, and there was a profuse discharge of glairy, semi-transparent matter. He was ordered a five-grain calomel pill, to be taken that evening; to follow it up with a draught, composed of inf. sennæ, in the morning, and to apply a small piece of lint to the sore.

Jan. 5to. The sore had slightly enlarged, extending towards the prepuce, and had become more hollow—its edges were hard to the touch, and the inflammation around became circumscribed—the discharge still continued equally profuse. He was placed upon the following treatment:

℞. *Pil. hyd.* ʒj. *Pulv. opii*, gr. iij. *in pilulas duodecim, sumat unam mane nocteque.*

To refrain from wine and beer, but not to confine himself wholly to the house. The sore to be washed twice a day with tepid water, and a small piece of lint to be applied to it. There was scarcely any alteration during the remainder of the month. About the 28th, there was greater difficulty in drawing back the prepuce, and he complained of severe shooting pain in the direction of the frænum. On examining it, three elevated, small, fungous sores were discovered at the edge of the prepuce, and on the body of the glans a hollow circular sore, differing in character from the others. The same plan of treatment was continued, and I directed the patient to syringe frequently during the day with warm water. On the 10th of February, he was seen by Mr. H. J. Johnson, when the mercurial fœtor was perceptible, and the appearance of the penis was nearly as just before described, having undergone very little alteration within the last fortnight.

He was then ordered to continue the blue pill, and occasionally to have a mild aperient—to take a wine-glass of the compound decoction of sarsaparilla twice a day—the *elevated* sores to be touched every third day with the nitrate of silver, and a piece of dressing to be inserted between the prepuce and glans, to absorb the discharge, which continued as profuse as at the commencement of the complaint. Under this treatment, the sores gradually assumed a healthy tendency, the hardness of the edges began to subside, and the application of the caustic reduced the fungous granulation to the level of the surrounding skin; the hollow sore of the glans became filled up by healthy granulations, and the constitution of the patient, which had suffered considerably during the exhibition of the mercury, has rallied again. On the 14th of March, three of the sores had completely healed, and the fourth, on the glans, is fast approaching to cicatrization."

These cases are sufficient to display the essential characteristics of the sore, and the influence of treatment. More might be added; but their sameness would tend rather to disgust the reader than to illustrate the subject. The complication with phymosis may be easily imagined, and, as no peculiarity is necessary in its management, examples may be readily dispensed with.

It was mentioned in the general account of the affection, that sometimes it commences in the form of an extensive vesication. Of this I have only seen two examples, and both are so interesting as to merit their narration.

CASE 4.—*Extensive Vesication in the Angle, ending in numerous Sores—Cure.*

I was summoned, last Autumn, to see a gentleman, aged about 21, who lived in the neighbourhood of the Lock Hospital, where I then resided as house-surgeon. The prepuce was inflamed and much swollen, hastening, apparently, with rapidity to phymosis. The angle presented throughout one extensive inflamed vesication, not containing serum, but dirty yellow lymph. It conveyed to the mind the impression that arsenic, or some acrid poison, had been applied to it and retained. The part was extremely painful.

There was an enlarged and painful gland in the left groin.

There was fever, with a loaded tongue.

He had slept with a stranger two nights previously. He had noticed the affection on the morning of the day on which he requested my attendance.

*Calomel at night, and senna, with the sulphate of magnesia, in the morning—saline medicines, with antimony—tepid ablution and bread and water poultices—repose in bed—starvation.*

This plan was continued for five or six days, when the bubo had *quite* disappeared, and the inflammatory swelling was gone. The cuticle of the angle, and the lymph beneath it, had separated from the cutis, leaving an extensive series of sores, very slightly cupped, more or less confluent, yellow on the surface, with abrupt, thin, and slightly excavated edge.

*Pil. hyd.* gr. v. *o. m.*
*Inf. ros.* c̄ *Mag. sul. o. m.*
*Lotio nigra ter die. Broth.*

In four or five days more, granulations had appeared, with the red line of cicatrization round the margin.

*Pil. hyd.* gr. v. *bis die.*
*Inf. ros.* c̄ *Mag. sul. o.* 3*tio mane.*
*Lot. rubra et Cer. calaminæ. Fish.*

In the course of a few days, the diet was augmented to mutton-chop for dinner; and the sores, which were now elevated, were touched with caustic.

In about three weeks from the commencement of the complaint, the sores were perfectly healed, and no induration of moment was left. His mouth was very gently affected. He was directed to continue the blue-pill for ten days. This he did, although he quitted town on an expedition to the coast in his yacht. On his return he took some sarsaparilla. His health has continued good, and he had no unpleasant consequence from the complaint to this time, March, 1834.

This case is extremely satisfactory. The prevention of impending bubo and phymosis—the rapid cicatrization of the sores, and the perfect nature of the cure, are strong evidence of the propriety of the treatment. It was the first case of the kind which I had witnessed, and it made a considerable impression on my mind.

The succeeding cases are intended to display the secondary symptoms that follow this species of sore.

Case 5.—*Plurality of Sores—cured without a Mercurial Course—Syphilitic Lepra.*

I have unfortunately lost my notes of this case. The circumstances are, therefore, presented from memory. They are accurate, though imperfect.

A young man was admitted into the hospital, under the care of Mr. Briggs. The prepuce naturally covered the glans, but it still admitted of retraction. He had sores on the inner prepuce and corona, of the nature of those in the former cases. He was treated by mere purgation and the antiphlogistic regimen. Caustic was also occasionally applied. The sores healed, though very slowly, and the patient left the hospital, after a sojourn of two or three months within its walls.

In a month or two after his dismissal, he returned one morning to shew himself to me, on account of an eruption. It was the circular, brownish-red blotch, flat, attended with very slight interstitial deposition in the cutis; in short, what has been termed the syphilitic lepra. The tonsils were vascular and thickened. It was evident that the patient had genuine secondary symptoms. I advised him to come into the house. He hesitated—went away—and we never subsequently saw him.

Case 6.—*Plurality of Sores on the Inner Prepuce—Combination of Roseola, Papula, and small Pustule—Mercury—Cure.*

Thomas Paradise, a servant, æt. 20, admitted July 11th, 1833, under the care of Mr. Briggs.

Several sores of the character of the preceding on the inner prepuce; thickening of the latter on the right side of the frænum, on which, at the angle, there is a flat, elevated ulcer. On the right side of the body of the penis, two recent ulcerations, yellowish, cupped. On the glans, one of these sores in its commencement; a small pustule, or turbid vesicle, beneath the epithelium.

Scrotum red, corrugated, with numerous pustules not yet ulcerated.

*Eruption.* A roseola-like efflorescence, disseminated on the chest. On the back and on the limbs, but chiefly on the former, an eruption of very small pustules, or of papulæ, pustulating at their apices. Some of these are fading, and leaving a small, brownish, cicatrix-like stain.*

*Throat.* Soft palate vascular and tumid—whitish-yellow, but not deep ulceration of the tonsils.

Gums tumid from mercury—health pretty good—pulse rather frequent and full.

*History.* This is a confused one.

A pimple appeared in the angle, on the right side of the frænum (where the present elevated sore exists), about five months ago. It formed a sore, which healed about six weeks ago.

The present ulcerations appeared three weeks ago.

The eruption was first noticed between two and three months ago. It has since continued.

The throat has been affected for one month.

The treatment hitherto has been this. Between two and three months ago he took six pills, which did not make his mouth sore. Three weeks ago he went to the Marylebone Dispensary, where he took 18 pills, and used the black wash.

*Infus. ros.* c̄ *Mag. sul.* ʒj. *bis die.*

*Lot. nigra. Therma. Broth.*

25th. Fresh sores are appearing—their surface is excavated and yellow. Tonsils more ulcerated. Feels very weak.

*Solut. quinæ,* c̄ *Tinct. cinch.* ʒj. *bis die.*

*Lot. rubra et Cer. calaminæ. Meat.*

Aug. 2d. Feels stronger. Ulcerations not healing—granulations and elevation in some—fresh ones still appearing.

*Infricetur Ung. hyd. fort.* ʒss. *o. n. P.* c̄ *Solut. quin.*

26th. He has continued the treatment without alteration. The sores are quite healed, but there are still elevated cicatrices, with induration. The eruption is fading fast. Mouth sore, but not too much so.*

Sept. 5th. Cicatrices almost healthy. Eruption quite gone—throat well—health good.

He continued the mercury for a week or ten days longer, when he was dismissed, cured.

CASE 6.—*Diffused Vesication in the Angle—Yellow Ulceration on the Tonsils—Spots of small Psoriasis upon the Body—Mercury—Cure.*†

Marmaduke Richardson, æt. 17, an upholsterer, admitted Aug. 11th, 1833, under the care of Mr. Briggs.

Surface of the inner prepuce at the angle, and contiguous corona of the glans covered with a sort of vesication, or something intermediate between it and slough. It is apparently produced by lymph effused between the cuticle and cutis. Behind the corona, on the left side, this has been separated, and left an elevated yellowish sore.

Slight bubo in the right groin.—Health good.

*History.* The complaint has existed for two or three months, and began soon after connexion. It commenced as "a

* The venereal pustule leaves a cicatrix, the papula only a stain. But often the papula pustulates at its summit. The casuist has then to determine, if the reliquiæ be or be not a cicatrix.

* The mercurialist and his opponent may inquire what is meant by this expression. The patient had *not* a spitting-pot, but his gums were tumid and whitish, and the salivary secretion was *rather* increased.

† This is an instance of the secondary symptoms that succeed the vesication ending in elevated sores. I have related a case of the primary affection occurring in the person of a gentleman, (Case 4.) That and the present are the only examples that have come under my observation.

white speck." He has not used any remedies, or consulted any surgeon.

*Infus. ros.* c̄ *Mag. sul. bis die.*

*Lot. nigra—Broth.*

26th. Case in statu quo.

*Pil. hyd.* gr. v. *o. n.*

*Rep. haust. semel die tantum.*

*Lot. rubra, &c.*

31st. Sores nearly healed.

Sept. 12th. Ulcerations healed, but induration left. Spots of yellow ulceration have appeared on the left tonsil —more general yellow ulceration on the right. It is deep in neither.

*Therma. Garg. boracis.*

*Omr. Haust. mag. sul.*

*Ordinary diet.*

Sept. 26th. An eruption of small circular, yellowish-brown, scaly spots has appeared on the various parts of the surface of the body.* The ulceration of the throat is healing.

*Infricetur ung. hyd.* ʒss. *o. n.*

*Omr. pil.*

Oct. 24th. He has pursued the plan of mercurial inunction. The ulceration of the throat has healed—the eruption has faded, disappeared, and left only a faint brown stain unaccompanied by scaliness. The mouth is and has been gently affected. The health is excellent.

In about ten days more the inunction was discontinued, the stain having subsided into a very faint yellowish tint, which at times was almost imperceptible. The tonsils had healed with some thickening, but the latter was passing away.

He was dismissed cured. He reapplied at the hospital on the 10th of last January with a fresh primary infection. He had continued free from any symptoms of the former malady.

These are all the cases of secondary symptoms from this form of sore it has fallen to my lot to witness. They prove that those secondary symptoms are not constant in their character, for, out of three cases, there was one example of lepra—one of papula—one of small psoriasis.

Perhaps I may be suffered to allude to another form of ulcer, which appears to possess some degree of affinity to the one I have described. I have called it in my note-book the EXCORIATION-SORE. The poverty of the nomenclature is probably too obvious.

It is usually observed upon the inner prepuce, immediately contiguous to the angle—sometimes it is seen at the angle itself—sometimes on the glans—not unfrequently on the outer prepuce, at a little distance from its free extremity. It may exist at the same time as separated sores, in all these situations. But this is comparatively rare. This sore may be single, or there may be several; when the latter is the case, they may or may not appear in succession.

The main feature of the sore is *not* that it is cupped or that it fungates, but that it conveys to the mind the idea of its being an excoriation. It is small and circular, situated merely on the surface of the cutis, and never, so far as I know, penetrating through it. Perhaps this result might follow irritating or injudicious treatment.

It commences, apparently, as a small red spot, scarcely exceeding the size of a pin's head. With the aid of a lens, the cuticle is observed to be raised in the centre, and a very minute vesication exists. In a few hours, or in one or two days, and the period is always brief, the elevated cuticle is abraded or absorbed, and the sore has assumed its distinctive character. To that character reference has been made already. The sore is minute, circular, superficial, wearing the aspect of an excoriation. The colour of its surface is commonly intermediate between yellow and red. The surrounding skin is frequently inflamed in a slight, but a perceptible degree.

The duration of the sore does not seem to be determinate. It heals by a small scab, but this may appear in the

* I have called this in my note-book the "small psoriasis," as it bears a more close resemblance to the psoriasis guttata of Willan and Bateman than to any other *named* eruption. It would be out of place here to detail the circumstances that prove this to be a genuine venereal eruption. It follows with much certainty the condylomatous sore.

course of a few days, or not for a much longer period.

The cicatrix is generally red—sometimes, when placed on the outer prepuce, indurated. Yet the induration is always slight.

I am not aware whether secondary symptoms do or do not succeed this sore. I am disposed to think that, in general, they would not; but circumstances seem to render it probable, that in some few instances they would. This is but conjecture.

The sore is occasionally concurrent with gonorrhœa, and those individuals appear most disposed to it, whose prepuce naturally covers the glans.

The treatment is doubtful, indeed experimental. Sometimes general remedies are successful—at others they are powerless, and mercury is necessary. Black wash is the most eligible local application, combined with an occasional employment of the lunar caustic.

The diagnosis between this and the former kind of sore is unattended with difficulty or with doubt. *That* is cupped in the first instance, is elevated afterwards—*this* is neither the one nor the other; *that* has all the features of an ulcer—*this* wears the aspect of excoriation; *that* heals, like a sore by granulation and by cicatrization—*this* disappears in a scab.

The distinction between it and the herpes præputialis is not so obvious, yet still it is commonly decisive. In the herpes præputialis, as figured and described by Bateman, the characters and the progress are extremely different. In order to place this in a clear point of view, a quotation from that author, containing his description, may not be deemed impertinent.

"The attention of the patient is attracted to the part by an extreme itching, with some sense of heat; and, on examining the prepuce, he finds one, or sometimes two, red patches, about the size of a silver penny, upon which are clustered five or six minute transparent vesicles, which, from their extreme tenuity, appear of the same red hue as the base on which they stand. In the course of twenty-four or thirty hours, the vesicles enlarge, and become of a milky hue, having lost their transparency; and on the third day, they are coherent, and assume an almost pustular appearance. If the eruption is seated within that part of the prepuce, which is in many individuals extended over the glans, so that the vesicles are kept constantly covered and moist (like those that occur in the throat), they commonly break about the fourth or fifth day, and form a small ulceration upon each patch. This discharges a little turbid serum, and has a white base, with a slight elevation at the edges; and by an inaccurate or inexperienced observer it may be readily mistaken for chancre; more especially if any escharotic has been applied to it, which produces much irritation, as well as a deep-seated hardness beneath the sore, such as is felt in true chancre. If no irritant be applied, the slight ulceration continues till the ninth or tenth day nearly unchanged, and then begins to heal; which process is completed by the twelfth, and the scabs fall off on the thirteenth or fourteenth day. 'An affection very similar in every respect sometimes occurs on the labia pudendi.'

When the patches occur, however, on the exterior portion of the prepuce, or where that part does not cover the glans, the duration of the eruption is shortened, and ulceration does not actually take place. The contents of the vesicles begin to dry about the sixth day, and soon form a small, hard, acuminated scab, under which, if it be not rubbed off, the part is entirely healed by the ninth or tenth day, after which the little indented scab is loosened, and falls out." *P.* 338, 3*d Edition.*

It must be owned that these sores have some affinity with the herpes præputialis. They occur, for the most part, in persons whose digestive organs are deranged, and who are subject to slight or chronic inflammation of the inner prepuce. On this account it is always proper to commence the treatment with the use of those measures which correct disorder of the organs of digestion, restore and improve the secretions, and benefit the health. But the cases I

shall introduce are sufficient to prove that this will not always effect a cure, and the cautious and judicious surgeon, who does not feel inclined to sacrifice his patient to his preconceived ideas, will sometimes be constrained to exhibit mercury.

CASE 1. *Excoriation-sore, healed in a few days.*

A medical pupil applied to me in the latter part of October, labouring under a state of excitement and alarm.

The inner prepuce was red and had an irritable aspect. On its left side were one or two small and circular sores, pale, quite flat, situated merely on the surface of the cutis, and conveying to my mind the idea of their being the result of excoriations.

The digestive and urinary organs were deranged. The gentleman was pallid and extremely nervous.

He had had these sores for four or five days, and they appeared very shortly after connexion. He had taken blue-pill once or twice daily, in consequence of his alarm.

I understood that he had had such sores before—that he had *"thrown in"* mercury—and that ulcerated buboes and protracted illness had resulted.

*I prescribed five grains of blue-pill every night. Infusion of roses and salts every morning. Low diet—repose—ablution and lint.*

In three or four days he returned. The sores were nearly healed—the irritable state of the inner prepuce was gone—the health was greatly improved. I diminished the quantity of blue-pill to three grains at night—prescribed a draught containing the carbonate and sulphate of magnesia in the morning—a lotion of sulphate of zinc—and better, but moderate diet.

He returned in the course of a week. The local complaint was well—the general health was comparatively good.

No other symptoms afterwards occurred.

CASE 2.—*Excoriation-sore, healed in a week.*

W. W——h, Esq. aged 21, applied to me on the 14th November last.

On the left side of the inner prepuce, near the angle, was a small superficial sore, as from excoriation. It was flat, yellowish, not cupped. The prepuce was slightly red around it.

He had had communication with a stranger on the 10th. He first observed the sore, which was extremely minute, on the 13th.

*Pil. hyd.* gr. v. *o. n. Infus. ros.* c̄ *Mag. sul. o. m. Lot. nigra.*

For two or three days the sore enlarged,* continuing flat, but exchanging its yellowish for a redder tint. The sore then began to diminish, and as moisture was applied to prevent a scab, it healed with a minute red border. The surrounding redness of the prepuce was removed. The bowels were open twice or thrice daily. There was no disturbance of the health.

*Lot. Bat. camph. vice lot. nigr.*

On the 21st the sore was healed, a healthy cicatrix being left, with, *perhaps*, a thickening of the rim.

*Pil. hyd.* gr. iij. *(vice* gr. v.*) o. n. Dec. sars. c.* Oss. *quotid. Omr. Inf. ros.*

Dec. 1st. Health good—no induration nor unpleasant appearance about the cicatrix. Mouth not perceptibly affected.

*Omr. pil. P.* c̄ *Sarsâ.*

He took the sarsaparilla for a week or ten days, when he finally discontinued medicine. His health was better than before he contracted the sore. He has since remained quite free from complaint.

CASE 3.—*Excoriation-sore resisting simple treatment—yielding to moderate doses of mercury.*

I was consulted in the Spring of last year by a tradesman residing in St. Martin's Lane. The inner prepuce was rather red, and on it was observed a circular sore, resembling those in the former case.

He had had this sore for ten days or a fortnight—had taken some aperient medicine, and applied some lint.

I ordered the blue-pill every night—

* At its utmost size, it did not equal a *very* small pea in dimensions.

the infusion of roses and salts every morning.

In a week the sore was not healed. The bowels were two much relaxed.

I continued the blue-pill—conjoined with it the decoction of sarsaparilla—and touched the sore with the lunar caustic.

In a week or ten days the sore was quite healed, and the cicatrix in this, as in the former case, presented no induration. The mouth was a little affected.

I directed the mercury to be discontinued;—the sarsa to be taken for a short time longer.

The patient has remained free from complaint.

CASE 4.—*Excoriation-sores, for which mercury was necessary and successful.*

Mr. W. Hering, of Foley Place, consulted me on the 26th of January last, on the case of a gentleman, who had been under his care, and in whose recovery he was warmly interested.*

The gentleman was about 19 years of age. He had gonorrhœa, with some scalding. There were several small circular sores, of the character frequently referred to, on the inner prepuce, chiefly at the angle. There was slight surrounding redness—scarcely any tumefaction, The gentleman was pale—the tongue was rather loaded.

About a fortnight previously he had connexion with an acquaintance, with whom he remained for some time.

In 48 hours he observed urethral discharge. In 12 hours more the sores of the inner prepuce had appeared. In 24 hours more there was ardor urinæ.

He took under Mr. Hering's direction purgative medicine and copaiba.

*Pil. hyd.* gr. iij. *Pulv. ipec.* gr. ij. *o.n.*
*Mist. mag. sul.* c̄ *Mag. carb. o. n.*
*Lot. nigra.*

In a week some of the sores were healed, but others had appeared, and, in fact, there was a marked disposition for fresh ones to start up. They were minute, yellowish, circular, insulated, looking as if the cuticle had just been picked out, with slight surrounding redness.

*Pil. hyd., o. a. n.*
*Mist. Magnes., o. a. m.*
*Lot. rubra. Argent. nit.*

In another week, very little progress had been made. Some sores were healed; others were established. There were two or three on the glans. One on the outer prepuce, just at its free edge, was larger and deeper than the rest, with something like induration of its basis. It had been formed by the confluence of two or three.

*All the sores touched every three or four days with caustic. P.*

*Feb. 15th.* The sore on the outer prepuce has displayed rather more induration; it is not healed—it is slightly irregular, of about the size of *half* a split-pea—yellowish on the surface with redness around. All the others which existed at the time of the last report are now cicatrized, one or two in the angle having left minute indurations. Two fresh sores had appeared on the dorsum of the glans, contiguous to the corona, of precisely the same character as all had evinced in their commencement. There was another on the glans near the orifice of the urethra; a slight scab was on it. The mouth was not sore. The gonorrhœal discharge was nearly gone.

I concluded that we had given a sufficient trial to general treatment. The patient had taken mercurial aperients for nearly three weeks, yet fresh sores still sprung up and ran the same course as their predecessors. The disposition to confluence, to the formation of a large sore, and to induration of its basis, strengthened, if it did not confirm my suspicions, and determined me to put the patient through a mild mercurial course. With the concurrence of my friend, Mr. Hering, who took the same view of the circumstances of the case, the young gentleman was placed upon the following plan.

*Pil. hyd.*, gr. iij. *bis die.*
*Decoct. sars. c.* ℥ij. *ter die.*

* I saw, in consultation with this gentleman to-day, (March 18th,) an interesting case of excoriation and paraphymosis. It was necessary to operate.

*Lotio nigra tantummodò.*

*Mist. magnes. sulph. o. 3tio. mane.*

*To take meat every other day—and to abstain from wine or beer.*

*March* 16*th*. The plan detailed above has been steadily pursued. In about a week from its commencement the sores had cicatrized, and no fresh ones have appeared. There is now scarcely any, even the most minute, induration left in the site of the ulcer of the outer prepuce. Yet there is still a slight degree of redness on this and on the inner prepuce, and as it naturally covers the glans in this gentleman, it is probable that neglect would still occasion recurrence of the complaint. The health is excellent, and he has gained both strength and flesh. The gums are very gently affected.

*To continue the pills for one week—the sarsaparilla for three. To retract the prepuce and use ablution with cold water thrice daily—and to keep lint constantly applied in the angle, and between the inner prepuce and the glans.*

This case exemplifies in a striking manner the observations which were made on the characters of the sore and the principles of treatment. The latter in this form of venereal ulcer should always be experimental. Mercurial purgatives or aperients, abstemiousness in diet, repose, ablution, the application of black-wash in the first instance, and caustic afterwards, should be tried in the initiative of every case. But, *if* the trial fails, I conceive that the surgeon must be wedded to opinion should he hesitate to prescribe a mild course of mercury. I might mention other cases in corroboration of this view. They would encumber what requires no further illustration.*

Another affection should be mentioned here, as it seems to enjoy some natural affinity to the excoriation sore. It is what may be denominated chronic inflammation of the inner prepuce. I am not aware that this has been mentioned by any author of consequence, indeed the descriptions contained in this paper are exclusively drawn from my own observation, uninfluenced by the opinions and experience of others. The affection in question is one of much interest and of some importance. In a gentleman on whom I am now in attendance, it has led to a separation from his wife for upwards of three years. But the length to which these remarks have extended, warns me to forbear inflicting more on the patience of my readers.

---

### Rochdale General Dispensary.*

### II. Report of Cases treated by Mr. Bower.

### 1. *Ascites. Paracentesis performed.—Cure.*

Thomas Mills, ætatis 18, of poor parents, and scrofulous habit, admitted March 20th, 1833, presenting the following symptoms and appearances.—Sight of left eye irrecoverably lost, from accident. Left elbow-joint much enlarged, with numerous sinuses discharging a thin unhealthy matter. Abdomen greatly distended, with evident fluctuation. Face pale and cachectic, tongue red, much thirst, appetite impaired, urine scanty and high coloured, bowels open, slight œdema of the lower extremities, great emaciation and debility.

States that he has worked in a factory from childhood. About two years ago a bobbin was thrown at him, which hit him on the eye, and instantly de-

* It is singular how readily this sore yields to ordinary treatment in some cases, and resists every thing but mercury in others. Yet the closest observation can detect no obvious difference of character. I once *saw* this sore treated off hand by mercury. The mineral was "thrown in." It is many months (they amount to years) since the gentleman contracted the malady and suffered the treatment. His health is broken for ever.

* This report has been forwarded for insertion in this Journal. Our anxiety to promote the publication and diffusion of clinical facts induces us to accede with pleasure to the proposal.—*Eds.*

prived him of sight; the globe appearing to have been ruptured, the humours escaping and the eye collapsed. A month after this accident his arm was violently twisted by an overlooker, and from that period he dates his present complaints.

He had been under the care of several practitioners in the neighbourhood, and taken various medicines without relief.

He was now ordered quinine with diuretics, friction to the lower extremities and abdomen, and the iodine ointment to the elbow-joint, generous diet, and moderate exercise in the open air.

May 12th. General health a little improved, but the hydropic symptoms much increased, the abdomen distended and appearing ready to burst. A large pouch had formed at the navel, remarkably thin and transparent, which, on attempting to get out of bed without assistance, was ruptured, and a little fluid escaped. Paracentesis was immediately performed, and twenty-four pints of a clear, straw-coloured fluid evacuated. Medicines, &c. to be continued.

May 30th. Abdomen has almost regained its former size, and there is a slight discharge from the umbilical aperture.—Urine in small quantity.—Bowels regular, appetite moderate. To continue.

June 14th. Abdomen much reduced in size, and fluctuation less perceptible; umbilical opening closed; urine in greater quantity and a paler colour; thirst less urgent; appetite improving. Bowels regular. To continue.

July 23d. Has gained flesh and strength rapidly since last report. Abdomen has nearly regained its natural size—no fluctuation perceptible, œdema of lower extremities entirely disappeared, urine in proper quantity, &c., and feels able to resume his occupation.—Elbow-joint still a little enlarged, with slight oozing from two or three small openings.

Ordered friction with iodine ointment to be continued, and to take tinct. iodine internally. Generous diet and moderate exercise. Discharged.

The dropsical symptoms in this case would seem to have been the result of extreme debility, arising from the nature of his occupation in a heated and unhealthy atmosphere, scrophulous diathesis, poor diet, &c., producing a want of tonicity in the exhalant vessels of the peritoneum, thereby allowing a greater quantity of serum to be poured out than the absorbents were able to take up; for it is remarkable that, after the fluid was evacuated, and as the general health improved, the accumulation which had again taken place was soon absorbed, and no disposition evinced for its re-production afterwards.

### 2. *Lumbago, with Metastasis to the Testicle.*

*Case* 2. John Williams, ætat. 52, of spare-habit, and nervous temperament, had been subject to severe attacks of lumbago and sciatica for the last twenty years. On the 27th October, 1833, after long exposure to wet and cold, was seized with severe lancinating pain in the loins, shooting down the back part of the thigh, in the course of the ischiatic nerve, as far as the calf of the leg, occurring in paroxysms, with intervals of from ten to fifteen minutes, and much aggravated on the slightest motion; no fever, vomiting, or pain in micturition; bowels habitually constipated. Ordered friction, with stimulating liniments, to the loins, and to take—

*Hydrargyri submur.* gr. iv. *Ext. colocynthidis,* gr. x. *statim.*

*Magnes sulph.* ʒvj. *Infus sennæ,* ℥vj. *M. Cyathum vinosum secundâ quâque horâ donec alvus respond.*

28th. Passed a restless night, pain exceedingly severe—bowels freely opened—urine in moderate quantity—pulse 90 and soft—tongue slightly furred—thirst. Ordered to keep in bed: friction with tartar-emetic liniment to the loins; bottles of hot water to the feet. To take five grains of Dover's powder every three hours; warm diluents; warm bath, and one grain of acet. morphine at bed-time.

29th. Restless night—pain still severe — profuse perspiration — bowels open—makes water freely—thirst—

tongue furred—pulse 85 and soft. Ordered Dover's powder, friction, warm-bath and acet. morph. to be continued.

30th. No sleep—still in much pain—perspiration profuse—pustular inflammation on the loins—bowels open—fever moderate. Ordered to continue warm-bath and acet. morphiæ, and to take the following draught every three hours:—

℞. *Tinct. colchici*, ʒss. *Spt. ætheris nitros* ʒss. *Magnes. sulph.* ʒj. *Mist. camphoræ*, ʒj. *Syr. rheados.* ℥j. *M.*

31st. Was called early this morning from his having been seized in the night with excruciating pain in the left testicle, which was found to be a little enlarged, exquisitely painful on pressure, and the scrotum of a deep purple colour. Had had no sleep—pain in the loins entirely gone, but still complains of numbness in the left thigh and leg—skin moist—bowels open—urine in moderate quantity—fever moderate. To continue the draughts, with warm bath and acet. morphine at bed-time—warm anodyne—fomentations to the testicle.

Nov. 1st. Slept a little. Testicle still more enlarged and very painful, the pain shooting up to the groin—spermatic cord a little thickened and tender—scrotum much discoloured. To continue.

2d. Symptoms much the same. To continue fomentations, with warm bath and morphine at bed-time, and to take pulv. colchici, gr. x. every four hours.

3d. Has slept four hours. Testicle somewhat reduced in size and less painful—scrotum regaining its natural colour—nausea—frequent loose stools—urine in considerable quantity. To continue fomentations, with morphine, at bed-time, and colchici, gr. v. every three hours.

4th. Passed a good night—testicle much reduced in size, and not painful, except when pressed upon—scrotum still less discoloured. To continue colchicum, &c.

From this date, the symptoms gradually subsided, a slight numbness of the left thigh and leg only remaining.

10th. Free from complaint. Discharged.

This case is strikingly illustrative of the migratory character of rheumatism, and of its disposition to attack fibrous structure, in whatever part of the body it may be found. It is probable that the tunica albuginea of the testis became the seat of the specific inflammation after its transmigration from the loins, in the same way as it is occasionally observed to attack the sclerotic coat of the eye, producing what is denominated rheumatic ophthalmia. General or local abstraction of blood was not employed; for it has been found that, excepting in young subjects of plethoric habit, and where the accompanying fever is of a purely inflammatory character, it has not only failed to alleviate the pain and other distressing symptoms, but has tended to superinduce such a degree of debility, as to render the attacks more frequent afterwards. Colchicum, in the form of tincture, though given in pretty large doses at short intervals, for three days, did not seem to have any influence over the disease; yet, in the form of powder, its effects became speedily observable, producing nausea, purging, increased secretion of urine, great diminution of pain, and general amelioration of all the more urgent symptoms.

Case 3.—*Tic Douloureux, treated with Carbonate of Iron.*

John Ashworth, ætat. 21, a stout, healthy young man, began to complain, ten days ago, of a sense of stiffness of the muscles of the left side of the face, with occasional darting pain, commencing at the root of the ear, thence radiating over the cheek as high as the foramen suborbitale, and downwards to the ala nasi, upper lip, gums, and teeth of the same side. He had been subjected to a variety of treatment, as leeches, blisters, sinapisms, opiates, purgation, &c. without relief. He had had three of the molar teeth extracted at different times, with no other effect than that of exasperating his symptoms.

1833, Sept. 10th. Admitted a patient at the Dispensary. The pain, which he describes as being of a burning, lancinating kind, comes on in paroxysms at irregular intervals, frequently as often as four or five times in an hour, continues about a minute, and then instantly ceases. During the paroxysm he shrieks out in agony, and rubs the upper lip and cheek violently with both hands; the tears gush from his eyes, and the saliva flows from his mouth in streams. The left side of his face appears red and a little swollen, probably from the violence of the friction he uses to deaden the acuteness of the pain. Has had no sleep for several nights—appetite moderate—bowels regular, &c. Ordered—

*Ferri carbon.* ʒj. *Pulv. cinnamon. comp.* gr. v. *every four hours.*

12th. Paroxysms equally severe, but with longer intermissions. Has had some sleep. To take—

*Ferri carbon.* ʒij. *ter in die.*

14th. Paroxysms less severe — of shorter duration, with longer intervals. To continue.

16th. Only two attacks since last report. To continue the iron twice a day.

19th. No return of pain—sleeps well, &c. To continue the iron once a day.

24th. Free from complaint, and has returned to his employment. Discharged.

*** The subject of the case mentioned in page 177 of our last number, where recovery took place after 12 tappings, has been seen a few days ago, by Dr. JOHNSON, through the kindness of Dr. DICKSON and Sir WILLIAM BURNETT. The gentleman, a Lieutenant in the Navy, is quite recovered, and all trace of enlargement in liver and spleen gone. He is now in pursuit of employment in His Majesty's Service again. The Case is exceedingly creditable to the professional skill of Dr. DICKSON of the Royal Naval Hospital at Plymouth. *J. J.*

*22nd March*, 1834.

---

### III. LANCASTER LUNATIC ASYLUM.

Mr. Davidson relates the following cases in our Liverpool contemporary, for March of this year.

*Case* 1. Th. Bullock, æt. 55, died Sept. 3d, 1831. The prominent symptoms were, profound affection of the locomotive muscles—ulcers of the sacrum and extremities—marasmus—occasional pyrexial excitement—complete fatuity—automatic life—frequent screaming.

*Dissection.* "Cranium very thick. Minute pisiform eminences disseminated over the mesial lines of the hemispheres. About a pint of pure serum in the cavity of the arachnoid. Œdema of the arachnoid elevated in the form of small bladders, containing limpid serum. White, thickened, mucous condition of the arachnoid, from subarachnoid effusion confined to the vertex and mesial margins of the hemispheres, and particularly in the intergyral spaces. Clouded, opaque, and thickened condition, from albuminous deposit on its inner surface, of that portion of the arachnoid unconnected with the pia mater, and situated behind the fourth ventricle and commissure of the optic nerves. Pia mater completely adherent throughout to the cortical substance, and, when raised, tearing up its outer layer, which was uniform in depth, and rather thicker than a wafer. Cortical substance in a state of ramollissement throughout, its whole depth of a lilac colour, and the torn surface granular, bloody, and resembling rotten fruit in consistence. Vessels of the cortical substance much dilated and tenacious. Small, bloody, punctuated extravasations, in the cortical substance throughout. Medullary substance rather soft, and pale in colour. About two ounces of pure serum in the ventricles. Small white granulations disseminated over the arachnoid of the ventricles, giving it a downy appearance; these were met with in greatest abundance over the arachnoid of the corpora striata and thalami. White pulpy ramollissement of the septum lucidum and fornix, of the lower part of the corpus callosum, and of the cerebral matter immediately surrounding the ventricles. This is one of the layers of the cortical substance, associated with softening, redness, intimate adherence of the pia mater to its surface, and other evidences of inflammation, and inflammatory irritation, are amongst the most common morbid appearances to be met with in old cases of insanity, and are attended either with general paralysis or profound dementia."—*Archives.*

Such cases are frequently met with in large lunatic asylums, and where the unfortunate patients are, more or less, deprived of muscular power as well as mental. This species of paralysis, Mr. Davidson avers, is invariably produced by a chronic inflammation, or its consequences, in the periphery of the brain, first affecting the tongue, and afterwards the face and extremities. Till an advanced period of the disease, the general health does not appear to suffer much, and the appetite is good—often voracious.

*Case* 2. E. Madden, ætat. 40, died Sept. 13th, 1831. The prominent symptoms were typhoid pyrexia—constant agitation, requiring coercion—gradual sinking of the powers of life, without coma or convulsions. Maniacal delirium, succeeded by low muttering and complete insensibility, closed the scene.

*Dissection.* " Dura mater much injected, adherent over the left hemisphere to the inner table; its serous lining infiltrated with blood, particularly over the left hemisphere. Effusion of about eight ounces of bloody purulent serum into the cavity of the arachnoid; *cerebral arachnoid covered at the convexities with a thick layer of consistent yellow pus, which completely obscured the convolutions;* and when superficially examined, conveyed the impression that a new, half-organized membrane had been formed upon the old, so great was its tenacity, and so completely detached was it from the flocculi floating in the sero-purulent secretion; white, thickened condition of the arachnoid very apparent over the convexities after the removal of the purulent layer above described; clouded, opaque, and thickened condition (in a trifling degree) of that portion of the arachnoid situated behind the fourth ventricle and commissure of the optic nerves; cortical substance of a reddish, lilac colour throughout, and in several places marbled, red, and softened: excessive red punctation of the medullary substance, bordering upon uniform redness; the cut surfaces presenting numerous bloooy points, which enlarged on exposure to the air for a few seconds, and gave a rosy tinge to the white substance. Veins and sinuses gorged with blood. Pia mater thickened, uniformly red, and highly injected throughout; bloody infiltration, about two inches long and an inch broad, where it lined the anfractuosities of the right middle lobe."

Several other cases are detailed by Mr. Davidson in the same Number of the Archives, to which we refer our readers, as valuable contributions to the pathology of insanity, and of cerebral affections generally.

To the above, we beg leave to add a short letter from Mr. Johnson, of Dublin, on the same subject, and published in the same journal.

" In complaince with your request, I am happy in communicating to you the following particulars, connected with the pathology of the brain; they are the results of about forty autopsies made in the Lancashire Lunatic Asylum by myself, during last Winter and Spring. I have no hesitation in allowing any use to be made of them which you please, because I have no wish to make out a case for this or that class of theorists; but, after very carefully observing and comparing together the appearances of the brain in such a number of cases, it is my firm and deliberate opinion, that deranged functions of that organ will be found accompanied very constantly by many of the following imperfections in its structure.

A faulty development of the convolutions, especially of those in the anterior lobes, as compared with the middle and posterior; a shallowness of the anfractuosities or sulci, between the convolutions, with a thin layer of the cortical substance, frequently, too, softened, and of a more dirty grey tinge than naturul. Cerebellum sometimes smaller, but more often larger than its just proportion to the cerebrum, presenting the same alterations, in colour and consistence, as the latter.

A congested and more or less inflamed state of the pia mater, proved by the unnatural quantity of blood contained in the minute and capillary vessels which ramify through it; by the condensation and apparent increase of its substance; by the adhesions which it had contracted with the substance of the brain; so inveterate that it will be found impossible, in many cases, to strip off the membrane without tearing at the same time the surface of the brain; these morbid conditions, in the severe cases, will be found to extend into the sulci; and by the formation of pus, attended of course with considerable breaking down of the neighbouring parts.

Considerable effusions at the base of the cranium, in the great cavity of the arachnoid, and, but more seldom, in the ventricles; very palpable thickening and opacity in the arachnoid; small albuminous deposits in various parts of it, which may be compared to portions of curdled milk: these white streaks will be found extending on each side of the longitudinal veins throughout its whole length, but especially on the vertex of the head, an inch or two anterior to the angle of the lambdoidal suture; this part I have so often observed to be the seat of the most evident disorganizations, that I beg particular attention to the fact. The perforated places at the base of the brain present these alterations in the arachnoid very remarkably.

Great vascularity and thickening of the dura mater, its glistening healthy appearance replaced by a livid colour, and drops of blood exuding in many places from its surface, which has often been found adhering closely to the inner table of the skull; with occasional clots of blood in the sinuses.

The white part of the cerebral mass, and the internal figurate parts, which form such important features in the ordinary anatomical descriptions, rarely present any morbid appearances appreciable to examination beyond a slight softening, and an occasional light rose-coloured blush, or a clayey yellowish tinge, instead of the natural white colour. I have, however, seen a large abscess extending throughout the whole of the interior of one hemisphere, holding several ounces of matter. In the same case, I found osseous deposits in the substance of the brain.

I have examined the brains of patients labouring under very various symptoms of cerebral disease, but I have been less successful in reconciling the particular form and intensity of each patient's case with the *post-mortem* appearances than in convincing myself that there are certain phenomena to be observed in insanity and cerebral affections, depending upon the conformation of the brain and upon the state of its vascular system, and that the organ and its investments are equally liable to local disorders with other parenchymatous, cellular, serous, and fibrous tissues. If this last proposition be granted, the apparent contradiction to it which I have freely stated just above may be accounted for, either by my own inability, at present, fully to detect every change which has taken place, or by changes which the parts may undergo after death, or by the well-known difference which exists between one constitution and another in their susceptibility to natural or morbid impressions.

In offering my humble testimony to the accuracy with which I am convinced that the valuable papers published by you, about two years ago, will be found to detail the important characteristics of insanity, I deeply regret that ill health should have prevented you from continuing those researches, and hope that you may soon have it in your power to prosecute them more fully.

Believe me to remain,
Dear Sir, Yours, very sincerely,
J. JOHNSON, A.B.
Trin. Coll. Dublin."

---

### IV. MR. GUTHRIE ON STRICTURE.

We quote the following clinical observations on the destruction of strictures by caustic, from a lecture of Mr. Guthrie's, published in a recent number of our contemporary, the London Medical and Surgical Journal.

"It is not my intention to enter into the history of the different kinds of caustic which have been employed by our predecessors in surgery, or of the methods of using them; you will find them related at length both in French and English writers, to whom I refer you. I shall confine my remarks to the methods at present adopted.

The caustics now used are two, the *potassa fusa* and the *argentum nitratum:* the first has been almost entirely abandoned with respect to the urethra, and the other has fallen into unmerited obloquy and consequent disuse. You must not, from this remark, suppose that I am going to advocate its restoration to practice, for I am going to do no such thing; it would not be prudent to do so, even if it were proper, for the prejudices of mankind have been so greatly and effectually excited upon this point, that they must be gradually allowed to subside; they will not admit of being taken by storm. Like most other prejudices they have some foundation in truth; and it was the *abuse* of the argentum nitratum and not the *use* of it which has given rise to them. When a surgeon of reputation cures a number of patients by a particular remedy or remedies, his professional character is gradually, nay, sometimes rapidly, augmented; more cases come under his observation, and many that are not susceptible of cure by the means he employs; he is nevertheless constrained, or nearly so, to use them; mischief ensues, alarm is excited; it soon spreads, is fomented by the adversaries of the plan, and a mode of practice, which is really successful in many cases, is often abandoned from prudential motives. I honestly confess I dare not say to a stranger, whatever his case may be, and however useful a few applications of the argentum nitratum might be, that I mean to use it. I dare not do so until after a few visits, and we become better acquainted, and have more confidence in each other, perhaps only after he sees that he does not make much progress. I should lose my patient if I did, who would go to another, and might be told it was the very worst thing in the world; an opinion he would not fail to repeat. Such is the prejudice against it among the younger men in London, that when a man says he has been cured by it, the remark is, how lucky you were to escape; I would not suffer any doctor to burn me for all the world. Nevertheless the argentum nitratum is a valuable remedy, when properly and carefully used in appropriate cases and not abused. It has been supposed, 1st. that the argentum nitratum takes off spasm and irritation; 2nd, that it can destroy a long and narrow stricture; 3d. that it affects a permanent cure. The concurrent testimony of all writers establishes the first fact, and it is now almost the only object expected to be attained from its use. It is, however, capable of doing more when properly and carefully applied; and at some future period, when the prejudice which has arisen against its use shall have passed away, it will again take its place with other means, as a very effective remedy in certain cases of stricture. That it is capable of making a passage for itself, through a long, narrow, and impassable stricture, which has become hard, gristly, and irregular, through time and repeated attacks of inflammation, I do not admit; and the attempt to do it, under such circumstances, has been frequently followed by the occurrence of great inflammation, the formation of abscesses, of fistulous openings in the perinæum, and between the urethra and the rectum, of inflammation and abscess of the prostate and the bladder, and of profuse bleedings which, with all or many of the preceding train of symptoms gradually lead the unhappy sufferer to the grave. That the cure is more permanent than by other means may in some cases be the fact, but on the whole it is doubtful; and Sir E. Home, the great advocate for its use, in his later years and publications, admitted the necessity for the occasional passage of a bougie, in order to prevent a return of the complaint. According to his method, a bougie, the size or nearly so of the passage, was to be armed with caustic; and if one of this kind is used, it ought to be armed when the bougie is made, and not introduced afterwards, and the distance from the orifice to the stricture having been ascertained and marked upon it, the caustic bougie should be oiled or greased, which is the best mode when caustic is used. A bougie of the full size, or nearly so, of the urethra, is first to be passed down to the stricture to clear the passage, and after a minute or two withdrawn, when the caustie bougie is to be passed, and the end or point, in which the caustic lies, and is barely exposed, is pressed against the stricture for the space of a minute. The first effect is to coagulate the mucous secretion of the part, forming with it a whitish soft substance, which has often been mistaken for a slough; the second is by its stimulus to relieve and remove the irritation existing on the surface of the stricture, so that the person often feels much easier after a slight application, makes water in a fuller stream, and is greatly surprised to find that the desire he suffered from to pass it every hour or two has been materially relieved. Sometimes, however, the effect is the reverse, and particularly where the application has been more severe, or the irritation has been of a nature not to be relieved by it. The part becomes more painful, the desire to make water greater, whilst the passage of it is altogether obstructed, or it passes by drops with great suffering, until, by fomentations, opiates, &c. the increase of irritation and inflammation has subsided. It acts, therefore, sometimes like a two-edged tool, and this has been another reason for its disuse, but it partakes on this point only of the property, which all other remedies have of doing the same, and the fact inculcates the necessity for great care and gentleness in its use, whenever it is had recourse to. When the application is steadily continued to the surface of the stricture for a minute or more, its continued effect is that of a caustic, viz. the partial destruction of the part, which injured a dead surface, must be thrown off by the usual processes of inflammation and ulcerative absorption separating it from the living part behind. Where the stricture is slight and thin, or narrow, this will in general be effected without much inconvenience, but when it is thick and hard it will not often be done easily, but, on the contrary, the inflammation will cause a greater thickening of the part, and the long train of evils I have alluded to already, if not prevented by proper means and a speedy abandonment of the practice. If, however, the operation should be fortunate enough to succeed, the separation of the slough in the diseased part is often marked by a paroxysm of fever, or the occurrence of an alarming hemorrhage. The rigors with which the febrile paroxysm commences are strongly marked, and I am sorry to say take place occasionally even before the slough separates for the last time. They frequently occur after every application, or every one which has been in the least severe, and in such cases forbid its continuance. They are dependent on a particular sympathy which exists between the urethra and the system at large, and will occur as readily from violence as from the application of any caustic whatever. I know a medical man who had suffered from Walcheren fever, and who almost invariably had a paroxysm whenever caustic was applied, or a large bougie was used so as to cause irritation; and the first paroxysm was always followed by others at regular intervals, so as to reproduce his Walcheren ague, which was only cured by the administration of bark, &c. in the usual way. This sometimes takes place in a less marked manner; and whenever the return of an irritation or pain is periodical and regular, quinine will in general, in combination with bark and opium, be found the best remedy, when exhibited between the periods of illness.

The hæmorrhages from the urethra are caused by the sloughs separating and leaving the cells of the corpus spongiosum exposed, or by the ulcerative process extending to some small vessel, the canal of which is partially opened. These, it is said, cease of themselves, although not until a great loss of blood has been frequently sustained, and it has been recommended to let the parts alone. I cannot give you any opinion formed from personal experience, as I have never seen one of these bleedings from caustic; but I conceive that they should be met and treated like hæmorrhages from the same place from other causes, which appear to me to be of a similar nature.

The most alarming hæmorrhages I have met with have been from common causes; and I will mention to you two of them of the most prominent kind, as they also point out the practice to be pursued in such cases."

---

### V. Hopital de Beaucaire.

*(Clinique of M. Bland.)*

### On the Fibrinous Polypi in the Cavities of the Heart.

Case 1.—*Dyspnœa, Palpitations. constant sense of Suffocation—Death.*

Peter Dupin, a soldier, 22 years of age, of a sanguineous temperament, and healthy constitution, had always enjoyed good health up to the 12th of December, when he was seized suddenly with a difficulty of breathing, and with a feeling of weight, or pressure in the region of the heart. The symptoms became daily worse, and he was admitted into the hospital on the 23d, or the eleventh day of the disease. The report then states, that he laboured under dyspnœa, great oppression, sharp pain over the heart, and palpitations. Auscultation discovered that the movements of the heart were exceedingly tumultuous, and that the bruits accompanying the contractions of the auricles, and those of the ventricles, were dull and stifled. The pulse was frequent, small, irregular, and unequal. The functions of the other organs of the body appeared to be quite normal. The case was recorded in the hospital books as one of acute carditis. A venesection and 12 leeches to the pericardiac region were ordered.

24th. The cardiac pain less severe; other symptoms unrelieved.

Half a grain of powdered foxglove to be taken every four hours.

25th. This morning the oppression was much aggravated; the face was livid and seemed to be injected with blood; and soon afterwards the whole of the body assumed the same purplish hue, which became darker towards the afternoon. At this time the agitation was truly dreadful; the poor patient struggling so violently for breath, that he shook his very bed with his efforts. But his strength could not long endure such suffering; he gradually became weaker; an apoplectic râle was heard in his throat; the limbs were icy cold and bedewed with a clammy perspiration; and he died at 6 o'clock, p. m.

*Autopsy 24 hours after death.* Face of a violet colour; integuments looked as if they were ecchymosed. Upon opening the chest, the lungs were found to be healthy;* the heart was perhaps a little enlarged; the veins on its surface were gorged with black blood, but the texture of the viscus when cut through was normal; the surface of the left auricle was livid; and when opened, this cavity was found to be blocked up by a polypus, or fibrinous concretion, as large as a hen's egg;—it passed through the auriculo-ventricular orifice into the cavity of the ventricle, where it became split into branches, which were interlaced with the columnæ carneæ, adhering to these very strongly. The cavities of the right side of the heart were full of black blood, part of which was coagulated. The other viscera of the body were sound.

*Remarks.* The preceding case of polypous concretion in the heart we consider to have been purely idiopathic, and not depending upon any lesion of its structure.

The apparent slight increase of its size was connected altogether with the distended state of its cavities. The most important features of the case are, the suddenness of the illness occurring in a subject previously so healthy and strong, that he had been able to continue his duties as a soldier up to the day on which he was seized; and the entire absence of any necroscopic lesion, except the state of the left auricle and ventricle of the heart.

Case 2.—*Cephalalgia, Anorexia, Vomiting, Diarrhœa, insensible Pulse, and Death.*

A young girl, six years of age, habitually pale and inactive, but free from any derangement of the organic functions, was seized on the morning of the 30th of November with head-ache and loss of appetite. After noon vomitings and diarrhœa came on, and all her energies seemed to be quite exhausted,

* About five ounces of a limpid serum were in the pericardium.

or paralysed. Towards the evening, the body became cold and discoloured all over, the lips were livid, and the eyes dull; her speech failed, and the pulse could not be felt; but all this time the breathing did not appear to be difficult or oppressed. She died at 11, p. m., about 15 hours from the commencement of her illness.

*Autopsy,* 24 *hours post-mortem.* Cerebrum and cerebellum healthy; about one ounce of serum beneath the latter: lungs sound and crepitant; a few ounces of serum in the pleuræ; the size and texture of the heart unaffected; but, upon cutting open its cavities, a firm yellowish polypus was found to occupy entirely the cavities of the right auricle and ventricle, and to extend for about an inch into the pulmonary artery, which was almost completely plugged up with it. The left auricle was empty; and the left ventricle contained only a small clot of blood. The other viscera were normal.

*Remarks.* In this curious case, the polypus occupied the right cavities of the heart. There can be very little doubt, that the concretion was formed during the few hours which immediately preceded dissolution; for early on the very morning when the patient was seized, she seemed to be in her accustomed health, and did not exhibit one symptom which could indicate any cardiac malady.

CASE 3.—*Malaise, Anasarca, Dyspnœa, Vomiting, Irregularity of Pulse, Shiverings, and Death.*

A young boy, five years of age, having in a childish frolic exposed himself alternately to heat and cold, became, in a day or two afterwards, feverish; the face was puffed up, and the legs were observed to be œdematous. On the following day, the infiltration had increased, and a distinct fluctuation was perceptible on tapping the abdomen.

All these symptoms were however speedily dissipated by a few doses of calomel and squills, along with a diuretic mixture, and the boy was pronounced well. But on the following evening he was seized quite suddenly with dyspnœa, vomiting, thirst, and occasional delirium; and when visited next morning, all these symptoms were found aggravated; the breathing was oppressed and extremely hurried, (amounting to 60 respirations in the minute); the pulse was frequent, small, and feeble; the face was pale; the lips livid; and the pupils dilated. Blisters, and a stimulating antispasmodic potion were ordered. At five o'clock in the afternoon, a general convulsive trembling of the body came on, and this was followed by a stiffness of every part, by trismus, hurried and stertorous breathing, &c. Death took place three hours afterwards.

*Autopsy.* Cerebral vessels gorged with blood; substance of the brain healthy; about one ounce of serosity at the basis, and a small quantity in the lateral ventricles; lungs crepitant and otherwise healthy; heart normal as to size and texture; the right ventricle completely occupied with a fibrinous concretion, which was partly of a firm consistence, and of a whitish colour, and partly soft, gelatinous, and tinged red, near to the orifice of the pulmonary artery. Abdominal viscera sound.

*Remarks.* In this, as in the preceding example, we have to notice the very sudden and unexpected invasion of alarming cardiac symptoms in a child, who had been previously quite free from them. True, indeed, there had been anasarca and ascites, a few days before, but these had been speedily removed, and the boy appeared to be altogether convalescent, when a violent dyspnœa set in; at the same time, his features became altered, and his lips livid; phenomena which too obviously announced some serious impediment to the free circulation of the blood. The subsequent delirium, apoplexy, and convulsive movements depened no doubt upon the disturbance of the course of this fluid through the encephalon; the absence, however, of any organic lesion of the brain shewed that the head symptoms were merely symptomatic; and the soundness of the respiratory organs completely exculpated them from having caused the fatal issue of the case.

These three cases, which we have reported, may be said to illustrate the rapid or acute formation of cardiac polypi; the following is adduced as a probable example of the more slow or chronic occurrence of the disease—we say "probable," for, unfortunately, the diagnosis was not verified by dissection.

Case 4.—*Dyspnœa—Lividity of the Face—Irregularity of the Pulse—Suffocation.*

A girl, aged nine years, lively, of a fresh colour, good constitution, and who had hitherto enjoyed entire health, became afflicted, about the beginning of November, with a difficulty of breathing, and, at the same time, the vermilion colour of her cheeks assumed a purplish hue. She began to lose flesh, the features shrunk considerably, and the eyes became heavy and dull. As the dyspnœa was gradually increasing, she was put under the care of M. Bland, on the fifteenth day after the commencement of her illness. The report at this time states, that the face was injected and of a violet tint; the lips livid; respiration hurried and oppressed; pulse frequent, small, irregular, and unequal; the stethoscope announced that there was no lesion of the lungs, but that the movements of the heart were tumultuous, and their bruits dull. The child's appetite, notwithstanding all this distress, was scarcely at all affected, and she would even engage in some of her usual exercises. The remedies which were used were of little avail; the disease evidently gained ground, and, after two months' sufferings, proved fatal, the patient seeming to be actually suffocated. The whole of the body had previously assumed a blueish tint.

*Remarks.* Our reason for supposing that the disease of this girl was not any organic lesion of the texture of the heart is, that in most cases of such lesions, the progress of the symptoms is rarely so rapid as in the example we have related; on the contrary, they are developed very gradually and slowly, exhibit intermissions of longer or shorter duration during their course, and advance to a fatal issue only after a long protracted illness. That the respiratory organs were not the seat of the disease, we think, may be inferred from the normal condition of their functions, indicated by the stethoscope; and, on the whole, the more probable explanation of all the phenomena of the case seems to be, that they owed their origin to some obstruction of the circulation through the heart, and that this obstruction was caused by the gradual formation of one or more polypous concretions.

With the view of illustrating the history of the very important disease, of which the four preceding cases may be viewed as characteristic examples, we have drawn together from different sources short notices of several others, recorded in works of acknowledged authority.

*Case* 5. Hoffman relates that a young man complained of a fixed, heavy pain in the left side of the chest, above the nipple, and that this was accompanied with anxiety, dyspnœa, palpitations of the heart, and irregularity of the pulse, and to these symptoms was added, in course of time, œdema of the lower limbs. He died in a short time, and Hoffman found, upon dissection, that the diagnosis which he had formed (viz. that there was a polypus within the heart), was quite accurate; for no structural lesion could be discovered, and the only morbid appearance was the existence of four fibrinous polypi in the cardiac cavities; two in the right ventricle, one in the left, and another in the aorta. The same author, in his "Opera Omnia," relates another very analogous case.

*Case* 6. A young man experienced great difficulty of breathing, a sensation of a trembling or stifled agitation of the heart, and the dread of suffocation whenever he lay on his right side. The symptoms were always relieved, by turning on his left side. He died, and, when the body was examined, a large polypous concretion was found within the right auricular cavity.

*Case* 7. A female suffered from pain in the region of the heart, occurring at intervals, and which was attributed by her physician to organic disease of the organ. The pain became more and more severe, and at length was constant, and finally caused her death. On dissection, a fleshy, black polypus, of the size of a walnut, was discovered in the left ventricle.—*J. Schenkins, Obs. Med. Rar.*

Rivière mentions the case of a man, who, after a sudden alarm, was seized immediately with a tremor of the heart* and with dyspnœa; his pulse became irregular and unequal, and he died shortly from suffocation. The heart and large vessels were found obstructed; in the left ventricle alone there were masses, in substance resembling that of the lungs; and the largest of these quite plugged up the orifice of the aorta.

Bonnetus, in his Sepulchretum, relates the history of a young girl, who was suddenly attacked with great oppression, most painful dyspnœa, and who, after lingering for three days in great agony, died, apparently suffocated. The right ventricle was completely obstructed with a large fibrinous polypus, which extended into the orifice of the pulmonary artery. Wepfer, in his treatise on apoplexy, records the case of a woman, who had long suffered from dyspnœa and palpitations of the heart, and in whose body, the only discoverable post-mortem morbid appearance was the presence of a polypus in the aorta. Friend tells us of a young man, who experienced violent palpitations of the heart after recovering from fever. This patient died suddenly, and, upon dissection, an immense concretion was found in the left ventricle of the heart. The formation of these polypi appears in some cases to be the work of a long time. A man, whose case may be found in Hoffman's works, having drank a quantity of cold beer, when he was heated, was seized with palpitations of the heart; these continued to recur with more or less severity for several years; he died at last from peripneumonia. An enormous concretion was discovered on dissection, occupying the pulmonary artery. The preceding are a few, out of a host of similar examples, which seem to us to prove that polypus of the heart ought to be regarded as an actual and an idiopathic disease. But they have not been able to satisfy the mind of Morgagni; for he considers the greater number of cardiac polypi as the result of, and, consequently, as formed posterior to, the extinction of life; and admits only a very few as genuine examples of the contrary. He acknowledges, however, that the blood may spontaneously coagulate in the blood-vessels, when the circulation is from any cause obstructed or arrested, also in aneurismatic swellings, and in the heart during the last moments of life, the cause being, in all these examples, the retardation of the blood's current; why not, therefore, at any period of life, when this state of the circulation is howsoever induced? We have only to suppose that a syncope lasts beyond a certain time, and it appears to us that the foundation of a cardiac polypus may be formed at any time, even in a person previously quite healthy. Probably, the greater tendency to such depositions in some than in other constitutions, may be connected with the different conditions of the blood itself in different systems, and in the same system at different periods. Be this as it may, we are forced, by the mass of affirmative evidence, to admit the corectness of Hoffman's position :—" Sunt autem polypi tum potissimum gravium morborum mortisque causæ, si quando mole suâ aucti, vel quod sæpius fit, a levi tam internâ, quam externâ causâ, de sede suâ dimoti, libero sanguinis circulo ex uno cordis ventriculo per pulmonum vasa in alterum obicem ponunt, eumque pervertunt, aut quando orificia vasorum penitus occludendo motum sanguinis vitalem omnino tollunt"—*t.* iii. *p.* 278; and to express our opinion, that many cases of sud-

* This tremor of the heart may be considered as analogous to the muscular trembling of old people. It depends upon atony, and consists in a number of imperfect and precipitate contractions. In the case of the heart being so affected, it must be incapable of emptying its contents.

denly supervening dyspnœa, of disturbance of the central organs of circulation, of rapidly-fatal asthmatic affections, and of palpitations of the heart, owe their true origin to the development of polypous concretions in the cavities of the heart.—*Révue Médicale.*

---

VI. Traumatic Tetanus.

A midshipman, on board of an Indiaman, got wounded in the left foot by treading on a rusty nail, 14th August. He kept his watch during a cold, rainy night, and, by eight o'clock next morning, presented symptoms of trismus. An opiate with camphor was exhibited, but the tetanus increased, and the limb was cold, and as he described it, dead and powerless. The pulse was 120, and his situation altogether alarming. It was proposed to the surgeon of the ship, by Dr. Murray, the narrator, to tie the posterior tibial nerve, and thus cut off communication with the wound. This was done, and although almost incapable of articulating a word the moment before, he immediately opened his mouth with an exclamation. His countenance presented a remarkable change for the better, and he said he felt life returning to his leg, The original wound was dilated, and a poultice applied. His bowels were now copiously opened, after which he fell into a sound sleep, which continued four hours. The tetanic symptoms, however, though mitigated, did not disappear, and it was deemed adviseable to bleed the patient to syncope, two days afterwards. By the 18th of August the tetanic symptoms had nearly disappeared, and he convalesced rapidly from this time. This case is related by Dr. Murray in ths sixth volume of the Calcutta Medical and Physical Transactions.

---

VII. Neuralgic Affections of Stumps.

The following case occurred in St. Bartholomew's Hospital, and is published in the Medical Gazette, No. 325, by Mr. Crookes, the house-surgeon.

"Sarah Slyfield, about eighteen years of age, was admitted into St. Bartholomew's in July last. She had undergone amputation of the right thigh in the country about nine months before her admission, for disease in the knee-joint, the effect of an injury received several years previously. When received into the hospital, she stated that the pain in the stump had come on within a few days of the removal of the limb; and that, although the part had been carefully examined, and means adopted for her relief, it had continued to increase, almost without intermission, up to the present time. Shortly after the wound had healed, six months after the operation, a small lump formed in the extremity of the stump, which was opened, and several pieces of dead bone extracted, but without affording any relief. Menstruation had commenced about the age of sixteen, and continued regularly up to the period of the operation, but has since only occurred once. Her person was stout, with a hale and somewhat bloated countenance, and she complained of nothing but severe and incessant pain in the stump. Examination of the part much increased her distress, as the slightest touch in any point of its integument gave her pain. The limb was naturally large from fat, but without any swelling or redness; and the extremity of the bone seemed well covered by soft parts. She was bled to sixteen ounces, and ordered purgative and saline diaphoretic medicines, and to apply linseed meal poultices.

This painful affection, which was so severe as almost entirely to prevent the patient getting rest, continued gradually to increase for about four months, in spite of the adoption of the most active remedies. Local and general bleeding, counter-irritation by moxa, narcotics, and the application of sedative plaisters to the stump, with tonics and antispasmodics, were all tried in vain; and the patient began to entreat to have the stump removed, to procure her, if possible, some alleviation of her sufferings. During this time she also experienced repeated attacks of hysteria; and was once seized with pain and acute sensibility of the integuments of the abdomen, which at first excited a suspicion of peritonitis.

As it was suggested by some that there might be suppuration in the extremity of the stump, a puncture was made into it, and the knife carried to a considerable depth, but without evacuating any matter, or producing any beneficial effect; it was therefore resolved to perform a second amputation of the limb.

*Operation.*—On account of the shortness of the stump it was impracticable to employ the tourniquet, and compression was made on the artery, as it passes beneath Poupart's ligament, by an instrument ordinarily used for that purpose. The muscles on the outer part of the thigh were first divided, and those on the inner, including the artery, last, in order that the vessel might be secured as soon as cut through. The bone being sawn through, and several small vessels tied, the ischiatic nerve was drawn out, and about an inch of it removed; some of the branches of the crural nerve were also cut shorter. Fifty minims of laudanum were given after she was taken to bed, as she was suffering great pain.

*Examination of the Stump.*—The extremity of the ischiatic nerve was bulbous, and of an almost cartilaginous hardness, and from this bulb there was continued in various directions a layer of dense cellular tissue, connecting it with the bone, the muscles, and the cicatrix. One of the filaments of the anterior crural nerve, perhaps the nervus saphenus, had also a bulbous termination, comparatively much larger than the ischiatic, and was similarly connected with the surrounding parts. The extremity of the bone appeared free from disease, nor was there any suppuration in its neighbourhood.

From the time of the operation up to the period of her discharge from the hospital, with the exception of a slight recurrence of the painful sensibility of the integuments of the abdomen, she continued to go on well, never having experienced any pain in the stump, or any unfavouable symptom. The remaining portion of the limb was very short, but sufficient to permit of the adaptation of the ordinary wooden leg, with the additional security of a strap to pass over the shoulder."

---

### VIII. Guy's Hospital.

### Clinical Remarks of Mr. Bransby Cooper on Injuries of the Head.*

Mr. Cooper has made some remarks on this interesting subject in the volume of his Clinical Observations lately published. It is a question which can never be exhausted, for the facts are infinitely varied, and scarcely less varied than complex.

The diagnosis between concussion and compression has exercised the ingenuity of able surgeons, and perplexed and confounded the minds of inexperienced ones. Some authors have related in graphic and perspicuous language the symptoms characteristic of either accident. The delighted student has closed the page, and proceeded with confidence to confirm at the bed-side its lucid information. He speedily finds that the volume of Nature is not so easily deciphered. Bewildered, disappointed, he turns with disgust from the ungrateful occupation, and either implicitly bows to authority, or smothers the doubts and the difficulties of reason in empirical experience.

Such are the phases of study, observation, scepticism, and despondency in the ordinary student of a hospital. He proceeds to practice hesitating and unsettled. His trust in dogmas has been shaken — his confidence in himself is not established. If active, industrious, and possessed of talent, a sufficient sphere of observation is presented to him, he gradually throws off the confusion which oppressed him, and emerges the practical experienced surgeon. We have probably sketched the progress of a Hey.

But the mental constitution of too many has never recovered the fever of

---

* Surgical Essays, &c. By B. B. Cooper, 1833.

doubt that seized it on exchanging the class-room for the bed-side. The malady has terminated in leaving the apothecary of after-life a confident sciolist, or a trembling practitioner.

Our text-books for the most part require revision—our lectures in many cases serve but to mislead. Errors and absurdities have passed as heir-looms from one generation of writers and teachers to another, and there are few who have published or have spoken the plain dictates of experience, the simple precepts of induction. Some may be excused for defective opportunity—more *could* only plead imperfect observation.

There is one surgical writer of modern times who stands pre-eminent for his philosophic labours. It is Mr. Brodie. Without pretension, with little art, his works are a noble specimen of unadorned inductive reasoning. Every opinion is the expression of facts, and none of his opinions are more. It is true that the limited powers of the human mind render even the inductive reasoner liable to error—it is probable that Mr. Brodie has not escaped the operation of this law of infirmity—but it is certain that the man who adopts the method he has chosen, will commit fewer blunders, and elicit more truth, than the copyist, the theorist, or the superficial reasoner.*

Mr. Cooper's volume is avowedly a record of facts, and of opinions which are only their expressions. The advantages of study which Mr. Cooper has enjoyed must necessarily induce us to regard his sentiments with attention and respect. We extract his description of concussion and compression.

"In cases of *concussion*, the patient is stunned, the pulse weak and fluttering, the face pale, and the extremities cold; these symptoms occur immediately after the accident, and they continue until a reaction commences, when a new train is presented. The patient now remains in a half comatose state, with his senses weakened but not lost, and his power of volition suspended but not destroyed. If he be addressed loudly by his name, he is capable of giving a rational answer, and when thus roused, his pulse is found to rise from its natural number to a hundred and twenty. In simple concussion, the pupils of the eyes have a natural appearance, and are capable of being stimulated by light,—nausea and even vomiting are frequently concomitant symptoms.

In *compression*, a complete comatose state comes on, and the senses and volition are entirely lost. The pulse is small, labouring, and hard; generally irregular, and sometimes intermitting. The pupils are dilated, and the retina insensible to light, occasionally one pupil will be dilated and the other contracted; and more rarely, both sides will be contracted. There is, however, no symptom, either in concussion or compression, more difficult to estimate as a diagnostic mark than the state of the pupil. I am, however, inclined to consider contraction of the pupils as an unfavourable symptom, portraying destruction of the nervous influence of the eye, and consequently great degree of injury to the brain. The breathing is stertorous. When the injury is very severe, hemiplegia is produced, and most frequently on the opposite side to that on which the injury has been received. These various symptoms may arise, either from the pressure of bone upon the brain, from extravasation of blood, effusion of serum, or from the formation of matter."

There are some points in this description to which we will take the liberty of alluding. Is Mr. Cooper certain that in simple concussion the pupils of the eyes have a natural appearance, and are under the influence of light? This does not accord with a succeeding sentiment—that the state of the pupil is an unsatisfactory diagnostic mark, both in concussion and compression. Neither does it accord with the observation of cases. We would say that in many instances, in which the circumstances rendered it probable that

---

* The works of Mr. Brodie on Diseases of the Joints, and on Injuries of the Brain, should be in the *memory* of every surgeon. Those who have complained that they are dull, have found that the study of nature is repulsive.

only "simple concussion" existed, the pupils were frequently dilated and insensible to the stimulus of light. We scarcely know what pure concussion is, for its symptoms mix so well and so often with those of slight compression and of injury to the texture of the brain, that it is difficult to determine where one ends and where the other begins. This consideration would be sufficient to make us pause in asserting that this or that condition of the pupil was essentially characteristic of concussion. When we turn to facts we find that in cases of what seems to be concussion, the state of the pupil is extremely various.

Mr. Cooper appears inclined to consider contraction of the pupils a very unfavourable symptom, and he states that it portrays "destruction of the nervous influence of the eye." That contracted pupil occurs in severe cases is indubitable, but we do not exactly see how it proves destruction of the nervous influence of the eye. Contraction of the iris does not imply paralysis of the organ so much as dilatation. When the belladonna is applied the pupil is dilated—so it is in amaurosis—so it often is in apoplexy, and in cases of compression where the retina seems totally insensible to light.

Mr. Cooper remarks that when the patient is recovering from concussion, the pulse will rise, when he is roused, from its natural number to a hundred and twenty. Of course this must be taken with reasonable exception—of course Mr. Cooper does not mean that this is absolutely, but only relatively true. Yet the student might be perplexed by the absolute assertion.

Mr. Cooper proceeds to mention that if compression is occasioned by the pressure of bone, the symptoms will come on immediately after the accident —but if from extravasation of blood or effusion of serum they are gradually established.

The latter observation is obviously imperfect. A patient receives a severe blow on the head. He is rendered senseless, continues so, has stertor, perhaps, and paralysis, and dies. There is found, on examination, effusion of blood on the surface, or at the basis of the brain. Here there has been no perceptible interval between the symptoms of concussion and compression; indeed it is most probable, that the blow which ruptured the vessel produced, at almost the same instant, both.

Mr. Cooper has omitted all mention of secondary hæmorrhage within the cranium, a circumstance of which the occurrence has been rendered certain by the observations of Mr. Brodie, and, we believe, of Dr. Macfarlane also.*

"Lastly,—when matter forms, days and even weeks may intervene, previous to the accession of the symptoms,—they are always preceded by a train of indications similar to the formation of matter in either parts of the body,—such as pains and rigors, after which, symptoms of compression occur, violent in proportion to the quickness and quantity of matter formed. Such symptoms lead to the necessity of surgical means for the removal of the matter. But a difficulty occurs to ascertain the precise point of the situation of the pus; puffiness of the scalp, easy separation of the pericranium, and the ashy color of the bone, form the marks by which the surgeon is authorised to use the trephine.

Too frequently, these symptoms come on insidiously, unattended with pain; but the surgeon should examine the scalp with care, when he will find the seat of injury still marked by a puffy state. Where the scalp has been wounded, the formation of matter may be more clearly pointed out. When this occurs, the edges of the wound, which were before healthy, will assume a glossy appearance, and the discharge becomes thin and ichorous."

Mr. Cooper has not noticed a fact of great importance, and not of extreme rarity. It is this. About the time when suppuration on or under the dura mater usually takes place, the symptoms that denote it may occur, and yet may be fallacious. Those symptoms are equally produced by suppuration

* See his volume of Clinical Reports.

within the cranium, and by the purulent deposites in the viscera, or in other portions of the body. If the surgeon is not aware of this circumstance, he may possibly be led to apply the trephine, when the mischief is seated remotely from the head. As an instance of the fact to which we have drawn attention, we may mention a case that occurred in St. George's Hospital. A man received some injury of the head, but we do not exactly recollect its nature. About ten days after the accident, he was attacked with rigors, succeeded by sweats. It was thought that there was suppuration within the cranium; but, no satisfactory evidence of its situation being present, the trephine was not applied. The patient sank. On inspection of the body, it was found that he had abscess of the liver.

"The inner table of the bones of the skull is sometimes fractured, while the external one remains entire: such an accident renders the diagnosis extremely difficult, because there are no external signs of the seat of the injury; or should there be any contusions of the exterior of the skull, still the fracture would probably be upon the opposite side, from what is termed a *contre coup*.

The effects of this accident would not come on until some time after the infliction of the injury, when effusion between the dura mater, and the inner table of the skull, would produce compression on the brain, and simultaneously a puffiness of the scalp externally, which would point out the seat of injury. In such a case, the dura mater is first separated, and the pericranium subsequently; the converse, however, more frequently happens, that the pericranium is first separated, and the dura mater subsequently; but, in either case, the train of symptoms which follow, are precisely the same."

We are not so sure as Mr. Cooper seems to be, that the fracture of the inner table would probably be on the opposite side to the stricken one. The well-attested instances of *contre-coup* are very rare—so much so, that it has been thought to occur only in those cases in which a blow upon the vertex occasions re-action on the basis, through the medium of the spinal column.

When Mr. Cooper states that the effects of this accident would not come on till some time after the infliction of the injury, we feel disposed to consider his language as loose—his description as deficient in particularity and exactness. We are tempted to inquire if Mr. Cooper alludes to depression as well as fracture of the inner table of the cranium. If not, we are inclined to doubt whether serous effusion on the dura mater must be looked on as a necessary consequence of mere fracture of that table. Whatever Mr. Cooper's meaning may be, we may probably lie open to the charge of misconceiving it. It certainly is not expressed with sufficient perspicuity.

"The treatment of both concussion and compression, are the same, so soon as reaction has taken place; which, however, is sometimes so slow in making its appearance, that it becomes necessary to employ stimuli, to restore the patient sufficiently, that he may be enabled to bear the means necessary to be employed. As soon as the pulse indicates the reaction, blood should be taken from either the jugular vein or the temporal artery, in such a quantity as may be considered expedient, regulated by the powers of the patient. A large dose of calomel, from eight to ten grains, should be immediately administered; which, if the patient cannot swallow, should be passed into the fauces upon a piece of butter, which is a better vehicle than any other, as it melts from the heat of the mouth, and allows the calomel from its gravity to find its way to the fauces. The head should be shaved, and evaporating lotions applied, a sinapism should also be applied to the soles of the feet, and small doses of the sulphate of magnesia given every hour until the bowels are freely opened. Should the symptoms not be relieved by the application of cold, a blister should be applied to the scalp. If all these means fail, under what circumstances is the trephine to be applied?"

We cannot approve, without reserve, Mr. Cooper's therapeutical directions.

It is not a little strange, that he makes no mention of general bleeding, in cases of inflammation of the encephalon after injury, and omits all reference to the influence of mercury, given to the extent of affecting the system. Yet active and judicious surgeons will bear us out in our opinion, that these are the two *most* powerful remedies at our disposal. When reaction commences, the patient should be bled and actively purged with calomel and senna. If inflammatory symptoms continue, the bleeding should be repeated again and again, the quantity and the frequency being determined by the decision and the prudence of the surgeon. We have bled a patient to incipient syncope twice within an hour or two under these circumstances, and we saved him. After the bleeding has been carried so far as is consistent with propriety, blisters should be placed upon the scalp, and dressed with the mercurial ointment. At the same time, the patient should take calomel, in order to procure, without loss of time, its effect upon the system. We are supposing a severe case; but our readers may be assured that such a case will not be brought to a favourable issue by inefficient or by temporizing treatment.

"If, attending these symptoms that I have described, there be a wound communicating with the bones of the cranium, accompanied with fracture and depression, or if there be fracture and depression, without a wound of the soft parts, or if there be puffiness of the scalp, either immediately after, or coming on, subsequently to the injury, then the surgeon is right in trephining; but, on the contrary, even if all these circumstances present themselves, but without the detailed symptoms, there is no evidence of the brain being injured, and therefore, I would recommend that the patient should be narrowly watched, and that the trephine should not be applied, until symptoms do point out the necessity."

It will be observed that Mr. Cooper differs from Sir Astley and from Mr. Brodie, in *not* recommending the employment of the trephine in cases of compound fracture of the cranium, attended with depression. We have not space for some interesting cases related by our author.

---

IX. MEDICAL REFORM.

The following passage from a powerful article in the Times of March 20th, will shew how the prospective reform in medicine is regarded beyond the pale of the profession.

"Parliament, as the result of the important inquiry which is now in progress, must prescribe the course of medical studies, or provide boards of medical examinations, independent of partial claims of rival corporations, and destructive of their despotic monopolies. A high standard of qualification must be fixed, which shall be equally applicable to the three kingdoms, and a conformable degree or diploma obtained in one, must entitle the licentiate to practise in all. There need, then, be no distinction made in the course of prescribed studies between the education of men whose object is to practise any branch of the medical art. Being qualified for all branches, those who have made themselves more particularly masters of surgery will devote themselves to the surgical department of the profession, while those whose taste or superior knowledge of diseases leads them to prescribe for their patients without operating, will become physicians, and both, as at present is the case with the general practitioner, will be permitted to dispense their own medicines. The profession would thus have the same kind of practitioners and the same division of labour, as hitherto; but it would derive its rights from a different source, — its classification would be formed more according to the natural order of things; — the usurpation of what was formerly its lower branch over the higher would be destroyed,— and no distinction of country, college, or corporation, would interfere with the legitimate exercise of professional capacity."

Let this passage be compared with our own sentiments on several late occasions.

